# Pseudohypoaldosteronism type 1: clinical features and management in infancy

**DOI:** 10.1530/EDM-13-0010

**Published:** 2013-08-30

**Authors:** N Amin, N S Alvi, J H Barth, H P Field, E Finlay, K Tyerman, S Frazer, G Savill, N P Wright, T Makaya, T Mushtaq

**Affiliations:** 1 Paediatric EndocrinologyLeeds Teaching HospitalsLeeds General Infirmary, LeedsUK; 2 Clinical ChemistryLeeds Teaching HospitalsLeedsUK; 3 Paediatric NephrologyLeeds Teaching HospitalsLeedsUK; 4 Paediatric MedicineBradford Teaching HospitalsBradfordUK; 5 Paediatric MedicineAiredale General HospitalKeighleyUK; 6 Paediatric EndocrinologySheffield Children's HospitalSheffieldUK

## Abstract

Type 1 pseudohypoaldosteronism (PHA) is a rare heterogeneous group of disorders characterised by resistance to aldosterone action. There is resultant salt wasting in the neonatal period, with hyperkalaemia and metabolic acidosis. Only after results confirm isolated resistance to aldosterone can the diagnosis of type 1 PHA be confidently made. Type 1 PHA can be further classified into i) renal type 1 (autosomal dominant (AD)) and ii) multiple target organ defect/systemic type 1 (autosomal recessive (AR)). The aim of this case series was to characterise the mode of presentation, management and short-term clinical outcomes of patients with PHA type 1. Case notes of newly diagnosed infants presenting with PHA type 1 were reviewed over a 5-year time period. Seven patients were diagnosed with PHA type 1. Initial presentation ranged from 4 to 28 days of age. Six had weight loss as a presenting feature. All subjects had hyperkalaemia, hyponatraemia, with elevated renin and aldosterone levels. Five patients have renal PHA type 1 and two patients have systemic PHA type, of whom one has had genetic testing to confirm the AR gene mutation on the *SCNN1A* gene. Renal PHA type 1 responds well to salt supplementation, whereas management of patients with systemic PHA type 1 proves more difficult as they are likely to get frequent episodes of electrolyte imbalance requiring urgent correction.

**Learning points:**

- Patients with type 1 PHA are likely to present in the neonatal period with hyponatraemia, hyperkalaemia and metabolic acidosis and can be diagnosed by the significantly elevated plasma renin activity and aldosterone levels.
- The differential diagnosis of type 1 PHA includes adrenal disorders such as adrenal hypoplasia and congenital adrenal hyperplasia; thus, adrenal function including cortisol levels, 17-hydroxyprogesterone and a urinary steroid profile are required. Secondary (transient) causes of PHA may be due to urinary tract infections or renal anomalies; thus, urine culture and renal ultrasound scan are required respectively.
- A differentiation between renal and systemic PHA type 1 may be made based on sodium requirements, ease of management of electrolyte imbalance, sweat test results and genetic testing.
- Management of renal PHA type 1 is with sodium supplementation, and requirements often decrease with age.
- Systemic PHA type 1 requires aggressive and intensive fluid and electrolyte management. Securing an enteral feeding route and i.v. access are essential to facilitate ongoing therapy.
- In this area of the UK, the incidence of AD PHA and AR PHA was calculated to be 1:66 000 and 1:166 000 respectively.

## Background

Type 1 pseudohypoaldosteronism (PHA) is a heterogeneous syndrome characterised by salt wasting resulting from target organ unresponsiveness to mineralocorticoids. As the principle effect of aldosterone is to promote potassium and hydrogen secretion, the condition typically presents in the neonatal period with hyperkalaemia, metabolic acidosis and an elevated plasma aldosterone concentration. Type 1 PHA can be further classified into two main clinical entities: renal PHA and systemic type/multiple target organ defects.

Renal type 1 PHA has an autosomal dominant (AD) pattern of inheritance with heterogeneous mutations on the gene coding for the mineralocorticoid receptor (1), resulting in an absence of binding of aldosterone to the receptor (2). The salt loss is restricted to the kidney, and biochemically, it is characterised by salt wasting, with hyponatraemia, hyperkalaemia and a metabolic acidosis, with elevated plasma renin and aldosterone levels. Although treatment is often straightforward with oral salt supplementation, the clinical expression of this condition can vary widely. It typically has a mild course, followed by spontaneous remission over time (3) (4). Over 50 different genotypes have been described, but there does not appear to be any significant correlation with clinical phenotype, and there may be variation in disease severity in patients with the same mutation (5).

The systemic form of type 1 PHA is an autosomal recessive (AR) disorder caused by mutations on the epithelial sodium channel (ENaC) (6) (7). In this form, symptoms are typically severe and persist into adulthood (8). There is defective sodium transport in many organs containing the ENaC, including the lung, kidney, colon, sweat and salivary glands. Consequently, sodium chloride is also elevated in sweat, saliva and stool (9) (10). Presentation is typically with salt-wasting episodes shortly after birth, and individuals can have an increased incidence of lower respiratory tract infections (10) (11). A positive saliva or sweat test showing increased sodium and chloride levels helps to complete the diagnostic picture for AR PHA (9) (10).

Owing to the rarity of the condition and transient nature of renal PHA type 1, the published literature has focussed mainly on case reports or case series (3) (4) (12) (13). The last reported case series in the UK was over three decades ago (3) and since then the demographics in parts of the population have significantly changed.

The aim of this study was to characterise the mode of presentation, management and short-term clinical outcomes of patients with PHA type 1 and for the first time calculate a clinical incidence for both renal PHA type 1 and systemic PHA type 1.

Patients with PHA were selected by searching the Supra-regional Assay (SAS) laboratory database for all elevated aldosterone concentrations (>5000 pmol/l) between January 2006 and December 2010. This was further corroborated by contacting all regional paediatric endocrinologists and nephrologists. The assays were performed in the SAS Service Steroid Centre, one of three national units performing aldosterone and renin assays for England. Aldosterone is analysed with an in-house RIA. Assays have been continuously monitored by external quality assurance schemes.

We retrospectively analysed the case notes of all children with confirmed PHA type 1. Secondary or transient forms of PHA (or type 3 PHA), which are associated with renal tract anomalies or urine tract infections, were excluded. The children were from Leeds Children's Hospital, Bradford Teaching Hospital and Sheffield Children's Hospital, UK. The initial presentation, management, biochemical evaluation and outcomes are documented.

## Case presentation

### Renal PHA type 1

In Cases 1–5, the diagnosis of renal PHA type 1 was made on the biochemical results and relatively mild disease severity.

#### Case 1

This male infant was born at term and presented at 3 weeks of life with vomiting and weight loss. Initial blood samples revealed hyponatraemia with a Na of 119 mmol/l. He was clinically stable and was initially managed for gastro-oesophageal reflux disease. When the plasma sodium failed to normalise after 6 days, a synacthen (1 μg) test was carried out, revealing a normal peak cortisol level of 1152 nmol/l. He was subsequently started on sodium supplements, at a dose of 3–5 mmol/kg per day, and fludrocortisone treatment was initiated. After receipt of his elevated aldosterone and normal 17-hydroxyprogesterone (17-OHP) levels, the fludrocortisone treatment was stopped and sodium supplementation was increased. His subsequent clinical course has been uneventful.

#### Case 2

This patient presented at 4 weeks of age with poor feeding, poor weight gain, hair loss and dry skin. He was found to be hyponatraemic and hyperkalaemic, with a metabolic acidosis on capillary blood gas sampling. Initial management was with i.v. fluids of 0.45% saline with 5% dextrose. He was started on oral sodium supplements, with a view to commencing mineralocorticoids if he became clinically unwell. After investigation, results indicated a diagnosis of PHA, his dose of sodium supplements was increased and low potassium formula milk was commenced.

#### Case 3

This female infant presented on day 8 of life with significant weight loss. Biochemical investigations revealed hyperkalaemia and hyponatraemia. Initial management involved sodium supplementation, hydrocortisone, fludrocortisone and calcium resonium. After the diagnosis of PHA was made, the hydrocortisone and fludrocortisone were stopped, and calcium resonium was gradually decreased. Subsequently, her management has been with sodium supplements alone.

#### Case 4

This male infant was born prematurely at 33 weeks gestation to a mother with diabetes. There was antenatal polyhydramnios, and at birth, the baby was macrosomic and hypoglycaemic. These findings were attributed to maternal diabetes. At day 4 of life, he was noted to have hypertension with macroscopic haematuria, which was thought to be secondary to prior umbilical artery catheterisation. The haematuria rapidly resolved and no renal abnormalities were identified. At day 10, the patient had profound hyponatraemia and hyperkalaemia, with a metabolic acidosis. A cortisol level at this time was 1070 nmol/l.

Initial management involved treatment of hyperkalaemia with salbutamol nebulisers and calcium resonium, administration of hydrocortisone and fludrocortisone, low potassium feeds and sodium supplementation. After establishing the diagnosis of PHA, the glucocorticoid and mineralocorticoid treatment was stopped and management has been with weaning doses of sodium supplementation.

#### Case 5

This normal term female infant presented at day 23 of age with profuse vomiting and failure to thrive, with an initial plasma sodium of 118 mmol/l and potassium of 7.3 mmol/l. She was managed on i.v. fluids, anti-reflux medications and an elemental milk formula. Plasma aldosterone and renin levels were elevated at 48 000 pmol/l and 16 nmol/l per h respectively. Subsequently, a diagnosis of AD PHA was made and sodium supplements were started. These were eventually weaned and discontinued and she was discharged from follow-up by 15 months of age.

### Systemic PHA type 1

Infants 6 and 7 have been diagnosed with systemic PHA type 1 due to the severity and longevity of electrolyte disturbance, positive sweat test results and systemic features of the condition. Patient 7 has had genetic testing which confirmed systemic PHA.

#### Case 6

This female infant presented at day 5 with jaundice and weight loss, after an uncomplicated pregnancy to consanguineous parents of Pakistani origin. She was admitted for establishment of feeds and developed progressively worsening hyponatraemia and hyperkalaemia by day 10. At this stage, her hyperkalaemia was managed with salbutamol nebulisers, and she was given low potassium feeds, i.v. hydrocortisone, sodium chloride supplements (3 mmol/kg per day) and sodium bicarbonate (1 mmol/kg per day). After 4 days of treatment, her electrolytes normalised, but 4 days later, hyperkalaemia redeveloped. After the diagnosis of PHA was confirmed, sodium supplements were increased, and mineralocorticoids were stopped. She has had recurrent episodes of weight loss, hyperkalaemia and i.v. fluid requirement necessitating an inpatient stay for many months. At 2 months of age, she required paediatric intensive care admission to manage an episode of severe hyperkalaemia.

A trial of treatment with sodium resonium was unsuccessful, and at the age of 2 years, she was started on indomethacin. This has helped to stabilise her urine output, reducing the need for i.v. fluids, and has allowed her to spend longer periods of time at home.

At times, she has required high doses of sodium chloride (up to 10 mmol/kg per day) and sodium bicarbonate (13 mmol/kg per day), without which she would have acute decompensation resulting in weight loss, irritability and hyperkalaemia. Significant reflux with gastrointestinal dysmotility and recurrent vomiting has resulted in poor oromotor skills. She had a gastrostomy inserted at 5 months of age, followed by fundoplication, but due to persistent retching, a jejunostomy was required at 6 months of age. She has had one episode of peritonitis at the age of 2 years, requiring maximal sodium supplementation in parenteral nutrition and i.v. 0.9% saline to maintain sodium concentrations.

#### Case 7

This male infant was born at full term to consanguineous Pakistani parents. He initially suffered from respiratory distress syndrome but was able to be discharged home after 2 days. At 10 days of age, he presented to hospital with a history of weight loss and drowsiness. Initial results revealed hyponatraemia, hyperkalaemia and a metabolic acidosis, which was managed as in Case 6. After 4 days of treatment, his electrolytes normalised, and hydrocortisone and fludrocortisone were stopped after a diagnosis of PHA was made. He was subsequently managed with sodium bicarbonate, sodium chloride supplementation and low potassium formula feeds.

He has had recurrent episodes of hyperkalaemia, requiring hospital admissions. The severity of electrolyte imbalance has resulted in episodes of cardiac arrest requiring resuscitation, and he has a central line *in situ* for emergency management. He has suffered from recurrent lower respiratory tract infections, and a sweat test was positive. He was therefore commenced on prophylactic flucloxacillin, with subsequent clinical improvement. He has had recurrent tonsillitis for which he receives prophylactic phenoxymethylpenicillin prior to a tonsillectomy.

Owing to difficulties feeding, nasogastric feeds were required prior to gastrostomy tube insertion. He has had recurrent episodes of vomiting and aspiration of feeds, eventually resulting in a fundoplication.

## Investigations

A summary of the cases with the initial biochemistry and treatment is presented in Table 1.

**Table 1:** Summary of cases. Plasma results of sodium, potassium, bicarbonate and pH are from the day of presentation, unless indicated. Initial urinary electrolytes, renin and aldosterone concentrations are from the initial admission

	**Case 1**	**Case 2**	**Case 3**	**Case 4**	**Case 5**	**Case 6**	**Case 7**
Age at presentation (days)	25	28	8	23	10	5	10
Symptoms	Vomiting and weight loss	Weight loss and poor feeding	Weight loss	Vomiting and weight loss	Inpatient	Jaundice and weight loss	Weight loss and drowsiness
Plasma Na (mmol/l) (RR 135–145)	119	126	118	118	109	142 (112 on D10)	126
Plasma K (mmol/l) (RR 3.5–5)	NA	6.8	8.2	7.3	6.8	6.1 (11.3 on D10)	10
Plasma HCO_3−_ (mmol/l) (RR 17–25)	19.4	17.9	19.6	NA	18.6	19.3 (D10)	10.4
pH	7.39	7.27	NA	NA	7.27	7.38	7.21
Creatinine (μmol/l) (RR 37–89)	67	61	29	48	96	74 (87 on D10)	49 (D11)
Urine K (mmol/l)	37	20	NA	19 (24)	10	22	7 (D15)
Urine Na (mmol/l)	50	16	15	20 (D24)	35	111	142 (D15)
Serum aldosterone (pmol/l) (RR 400–3000)	35 700	39 900	83 390	48 000	49 500	57 000	16 580
PRA (nmol/l per h) (RR 1.9–29)	41.3	250	>250	16	>250	>250	131.5
Duration of follow-up (months)	62	38	25	14	36	39	42
Weight centile at last clinic appointment	91st–98th	9th–25th	25th	50th	91st–98th	9th	75th
Ongoing management	Initial Na supplementation 2.5 mmol/kg per day	Initial Na supplementation 1.5 mmol/kg per day	Initial Na supplementation 2 mmol/kg per day	Discharged from follow-up. Not on medication	Initial Na supplementation 4 mmol/kg per day	Initial Na supplementation 3.8 mmol/kg per day*	Na supplements 8.5 mmol/kg per day*
	Now on 0.7 mmol/kg per day	Now on Na 0.7 mmol/kg per day			Now on Na 0.8 mmol/kg per day	NaHCO_3_ 1.6 mmol/kg per day*	NaHCO_3_ 2 mmol/kg per day*
						Indomethacin 1.5 mg/kg b.d.	Flucloxacillin 125 mg b.d.
						Gaviscon	Penicillin V 125 mg b.d.
						Ranitidine	Omeprazole 0.6 mg/kg b.d.
						Low potassium feeds	Domperidone 0.35 mg/kg b.d.
						Intermittent requirement for i.v. 0.9% NaCl	Montelukast 4 mg o.d.
							Beclometasone 200 μg b.d.
							Sodium cromoglycate 5 mg q.d.s.
Low potassium feeds
PRA (nmol/l per h) after long-term management	6.1	24.9	NA	7.4	1.1	0.3	Not done
Aldosterone (pmol/l) after long-term management	1230	5450	23.3	3400	385	<55	Not done
Renal and bladder USS	Normal	Normal	Normal	Normal	Normal	Normal	Normal

RR, reference range (age appropriate); PRA, plasma renin activity; NA, not available; o.d., once daily; b.d., twice daily; q.d.s., four times daily; USS, ultrasound scan.

* Doses subject to variation.

### Renal PHA type 1

Cases 1–5 presented with significant hyponatraemia and hyperkalaemia. The diagnosis of PHA type 1 was made after receipt of their significantly elevated aldosterone levels (ranging from 35 700 to 83 390 pmol/l) and increased plasma renin activity (ranging from 16 to >250 nmol/l per h).

Differential diagnoses based on the initial electrolytes includes hypoaldosteronism, secondary PHA, adrenal hypoplasia, congenital adrenal hyperplasia (CAH) due to 21-hydroxylase deficiency, or specific hormonal blocks within the adrenal biosynthetic pathway. Some of these enzyme deficiencies may be associated with indeterminate genitalia and consequently a range of biochemical abnormalities; neither of which were apparent in our infants. A detailed discussion of these is beyond the scope of this report.

All five cases of renal type 1 PHA had normal 17-OHP levels, renal ultrasound scans, sterile urine cultures, and a urinary steroid profile, thus excluding 21-hydroxylase deficiency or disorders of the adrenal biosynthetic pathway. Owing to the ease of management with good long-term outcomes, it was not possible to justify genetic testing for the children with renal type PHA.

### Systemic PHA type 1

Cases 6 and 7 had hyponatraemia and hyperkalaemia; however, in Case 6, this only became evident by day 10. Both patients had elevated plasma aldosterone levels and plasma renin activity (Table 1), with normal 17-OHP, urine steroid profiles, urine cultures and renal ultrasound scans. Both patients have had positive sweat tests, which is a commonly found feature of patients with AR PHA type 1. This indicates a defect in the ENaC that is present in many organs including sweat glands. Only Case 7 has had genetic testing, which revealed an AR gene mutation on the *SCNN1A* gene.

## Treatment

### Renal PHA type 1

Patients 1–5 have been successfully treated with sodium chloride supplementation alone. The dose of this has varied, with initial requirements between 1.5 and 4 mmol/kg per day. Sodium supplementation has reduced with time with one patient not requiring any supplementation by a year of age.

### Systemic PHA type 1

Patients 6 and 7 have required varying sodium supplementation with sodium chloride and sodium bicarbonate, and Patient 6 continues to have an intermittent requirement for i.v. 0.9% sodium chloride. Both patients have low potassium feeds, yet hyperkalaemia has remained an ongoing problem.

Ion exchange resins were utilised for the treatment of hyperkalaemia. Calcium resonium binds to potassium in the gastrointestinal tracts, thus limiting its absorption, whereas sodium resonium is another cation exchange resin that swaps sodium ions for potassium ions. Its use should result in a simultaneous correction of both hyponatraemia and hyperkalaemia. However, Patient 6 did not respond to a trial of sodium resonium unlike the reported cases with AR PHA (12) (13). Therefore, indomethacin was utilised due to its inhibition of prostaglandin synthesis, which results in a decrease in urine output and a reduction in renal sodium losses (14). The successful use of indomethacin in systemic PHA to help to regulate fluid balance has been previously described (15) (16), and in Patient 6, this has helped to stabilise the electrolytes and allow longer periods of time at home.

Owing to recurrent bacterial infections resulting in clinical decompensation and electrolyte imbalance, Patient 7 also received prophylactic antibiotics with a reduction in infection frequency.

Both patients with systemic PHA type 1 have had significant difficulties with enteral feeding, with severe gastro-oesophageal reflux requiring early fundoplication with gastrostomy or jejunostomy.

## Outcome and follow-up

### Renal PHA type 1

Our follow-up for Patients 1–5 ranges from 14 to 62 months. They are all thriving and remain on low doses of sodium supplements, apart from one case in which treatment has been discontinued.

### Systemic PHA type 1

At the age of 3 years, Patient 6 has had longer periods of time at home and she is currently on treatment with sodium bicarbonate, sodium chloride, indomethacin, gaviscon and ranitidine. She remains on full jejunostomy feeds with low potassium formula milk. Long-term i.v. access remains *in situ*. She continues to have regular episodes of hyperkalaemia and acute decompensation, often precipitated by intercurrent infections requiring hospital admission and management with i.v. fluids. Despite her positive sweat test, she has not had frequent or severe respiratory tract infections.

Patient 7 is now 42 months of age, and over the last year, the number of admissions into hospital with severe hyperkalaemia has significantly decreased. He continues to have recurrent central line infections requiring regular assessments as an inpatient. He receives weekly blood testing at home and remains gastrostomy fed.

## Discussion

PHA type 1 is a condition that may present with mild or profound salt wasting with hyperkalaemia.

The initial biochemical picture is similar to adrenal hypoplasia, CAH or secondary PHA and investigations should include plasma renin activity, aldosterone, 17-OHP, cortisol levels, renal ultrasound scan, urine cultures and a urine steroid profile.

Sodium and potassium homeostasis are regulated by the effect of aldosterone on epithelial cells in the collecting duct of the nephron (17). Aldosterone passively crosses the epithelial cell membrane and binds to the mineralocorticoid receptor. The ligand-bound receptor translocates into the nucleus, thus promoting or repressing gene signalling (18). Transcription of signalling factors results in an accumulation of ENaC at the plasma membrane, enhancing sodium transport into the epithelial cell (19). Sodium is then actively extruded out of the cell by the sodium–potassium ATPase on the basolateral membrane of the cell (20). Inactivating mutations in the mineralocorticoid receptor cause renal PHA type 1 whereas inactivating mutations in the ENaC subunit genes are the cause of the more severe systemic form of the disease (6) (7) (21). In this case series, five infants have the clinical appearance of renal PHA type 1 and two infants have systemic PHA type 1.

AD PHA type 1 is more common, and indeed five of the infants had this diagnosis. In most cases, patients presented in the early neonatal period with weight loss and biochemical analysis showed hyperkalaemia and hyponatraemia, with a variable metabolic acidosis. A negative sweat test would further differentiate these patients from AR PHA1. Treatment in AD PHA type 1 is with sodium supplementation, which generally becomes unnecessary by 1–3 years of age (4) (22). This improvement may be explained by the maturation of the renal salt conservation ability. In our group, the initial sodium requirements were 2–4 mmol/kg per day with little need for any increase with age. Cases 2, 3 and 4 did not appear to have significantly elevated urinary sodium losses, but the values are not dissimilar to those previously reported in a case series of children who had genetic confirmation of AD PHA1 (4).

The literature describes a wide clinical spectrum, with some patients apparently unaffected, others with elevated renin activity and aldosterone concentrations but normal electrolytes, and others with clinically significant renal salt loss (5) (23). In adults, elevated aldosterone concentrations may be the only biochemical marker of renal PHA type 1 (1).

Our patients with AR PHA type 1 had a much more difficult clinical course, requiring intensive support for stabilisation of electrolyte imbalances and feeding. Infant 7 has a confirmed gene mutation on the *SCNN1A* gene. Homozygous mutations in the SCNN1A gene in patients with AR PHA type 1 has been previously described (6) and result in loss of ENaC activity. Other gene mutations that have been described are mutations on the *SCNN1B* gene (6) or the *SCNN1G* gene (7).

Both patients have had positive sweat tests, helping to confirm the diagnosis of systemic PHA type 1. Unless clinicians are aware of this association, a positive sweat test may result in an incorrect diagnosis of cystic fibrosis, as both disorders cause excessive salt loss from sweat glands.

Unlike previous reports (5) (6), treatment with sodium resonium was not successful in Patient 6, whereas the introduction of indomethacin has helped reduce urine output, stabilise electrolytes and regulate fluid balance.

Case 7 had neonatal respiratory distress syndrome at birth, which is the first time this has been described in a term infant with AR PHA type 1. He has subsequently had recurrent lower respiratory tract infections. In AR PHA type 1, patients typically demonstrate a chronic pulmonary syndrome, characterised by recurrent coughing and wheezing without identifiable bacterial airway infection (11) (24). This is thought to be due to reduced sodium-dependent liquid absorption due to inactivation of ENaC in the pulmonary epithelium (9).

Management of AR PHA type 1 is with extensive salt supplementation, and vigilance for the systemic features of the disease, such as the respiratory complications. Despite close monitoring of these patients, the speed and severity of the electrolyte imbalance in Patient 7 led to a number of cardiac arrests requiring resuscitation, and Patient 6 has required intensive care admission due to severe hyperkalaemia. Gastrostomy/jejunostomy feeding and secure venous access have been crucial in the management of these two patients, and this is something we would recommend obtaining early after a diagnosis of AR PHA has been secured. AR PHA type 1 requires lifelong salt supplementation, but both of our patients have shown some improvement in clinical severity over time.

Other phenotypic features of patients with AR PHA type 1 can occur as a consequence of ENaC expression in other tissues. These include cholelithiasis (25), polyhydramnios (26) and characteristic skin changes (9), but these have not been apparent in either of our patients.

It is important to differentiate patients with systemic PHA type 1 from those with renal PHA type 1 as these patients are more likely to severely decompensate even with apparently trivial symptoms. It should be noted from our results that normalisation of PRA and aldosterone on treatment cannot be used as an indication for or against the genetic subtype of PHA. A positive sweat test may help endorse a diagnosis of AR PHA and genetic testing can be confirmatory.

### Incidence

There were found to be seven cases of confirmed PHA type 1 over a 5-year period. Using 2009 data from the office of national statistics (www.statistics.gov.uk/statbase, accessed 09/07/2011), the birth rate in the Yorkshire and Humber area was found to be 66 400 per year. Thus, the calculated incidence of PHA type 1 was 1:47 000, with the incidence of AD PHA and AR PHA calculated at 1:66 000 and 1:166 000 respectively. It is possible that some infants may not survive the initial salt-wasting episode or may be misclassified as adrenal hypoplasia, CAH or sudden neonatal death. Similarly, AD PHA type 1 may be underdiagnosed as it typically runs a much milder course and is easily treated with sodium supplementation.

## Patient consent

Patient consent has been obtained for the two patients with AR PHA type 1, as these patients may be identified from the report.

## Author contribution statement

J H Barth and H P Field gave inputs for the biochemical analysis and were involved in writing the report. Physicians: N P Wright, T Makaya, S Frazer, E Finlay, K Tyerman, G Savill, N S Alvi and T Mushtaq are responsible for patients.
